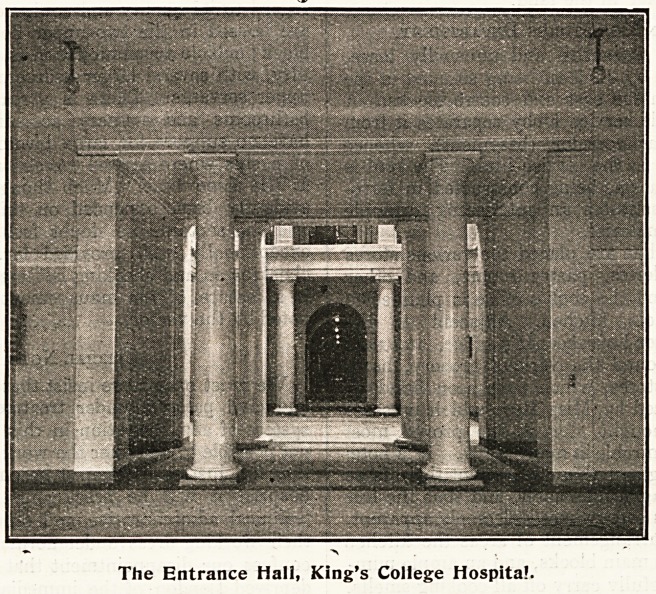# Educational Value of Mr. Pite's Creation

**Published:** 1915-12-18

**Authors:** 


### EDUCATIONAL VALUE OF MR. PITE'S CREATION.

We have briefly described the general scheme of the new King's College Hospital, and have indicated the position of the buildings and the purposes to which they will be put, a description which will be helped by a reference to the block plan given on this page. We now propose to run through a few of the departments and of sections of the accommodation, so far as it is possible to judge them in their working dress under existing conditions. Several of the departments cannot be so treated, and those portions of Mr. Pite's scheme to which they relate must be left for judgment in the future, when the whole establishment has become a civil hospital and every portion of his new buildings is at last working and organised to fulfil the purposes for which it was created. It will suffice for our immediate purpose, as an indication of what a centre of valuable information and instruction the new King's College Hospital in fact is, to confine ourselves to-day to a terse description giving a general idea of some most interesting features to be met with in connection with (1) the wards and special ward units; (2) the organisation of the administrative section of the buildings devoted to (*a*) the nursing department and its teaching-school, (*b*) the accommodation for (i) the matron, (ii) the sisters, (iii) the nurses, and (iv) the wardmaids; (3) the kitchen and stores departments.

### The Plan as a Whole.

One notable feature in Mr. Pite's plan is that everywhere, from basement to roof, Mr. Pite has so well carried out his ideals that he has secured an abundance of fresh air for every portion of the buildings and for every store and room, passage-way and corner which it contains. A visitor, who has been accustomed to hospitals and great blocks of institutional buildings, must be continuously struck by the wonderful efficiency of the system of ventilation secured throughout the new King's College Hospital buildings, many of the excellences of which are undoubtedly due to the thought and care expended upon the planning and situation of the buildings throughout. One small point we noticed which is a constant want in hospital buildings everywhere, *i.e.*, the omission to provide an inlet for air at the floor level of those sections of the stores, linen and other departments where a

(*Continued on page* 249.)

### EDUCATIONAL VALUE OF MR. PITE'S CREATION *(continued from p. 247).*

constant interchange of air from top to bottom is essential to secure the maintenance of an atmosphere which can never be dense enough to enable one, figuratively, to cut patterns in it with a pair of scissors. We know this is a detail, but it is a detail which has been fought for and insisted upon for forty years without making the slightest impression, apparently, upon the best of architects, though its paramount importance and necessity is patent if efficiency be the first consideration in planning the details of essential portions of every hospital building which aims at being as completely perfect as possible.

### Mr. Pite's Ward Units.

We fancy Mr. Pite must have studied the latest specimens of new hospital construction in Scotland. At any rate, consciously or unconsciously, fate has decided that instead of using the day room provided for patients for this purpose it is being used at Denmark Hill at present as a waiting-room for patients, which is one of the features of the Scottish system. The ward pavilions have their axes approximately north and south, a plan devised to provide the maximum amount of sunlight and to obviate any discomfort from the midsummer meridian. The main ward blocks are of three storeys. There will ultimately be nine pavilions, the spaces between each being sufficient to ensure ample lighting, that between the third storey and a two-storey block being sufficient to provide a full-sized tennis court. The roofs of the wards are flat and covered with asphalt. The sanitary towers are roofed with Italian tiles, and so are the tank houses on each three-storey block. There are sun balconies at the end of each ward which provide an uninterrupted view, and in warm weather have been used as outdoor dormitories. These balconies have the advantage of being screened from inclement winds by the flanking sanitary towers. Each of the lower balconies is well sheltered, those on the top storey being covered by a glass roof. A large-mesh wire netting is used as a protection against accidents, and access is given to the fire-escape stairs through the cut-off lobbies of the sanitary towers.

Taking the ward unit and approaching it along the main ward corridor, the visitor will note a wide arched opening leading directly from the main corridor into that of the ward, the latter being eight feet in width. Three pairs of swing doors are placed in this corridor, the first two on either side of the cut-off lobby and the third leading directly into the ward. On the left of the archway as the cut-off lobby is entered is the sisters' room, with a nurses' lavatory adjacent, on the right is the patients' day room, and in the same lobby there are a sink room and the wardmaids' closet. Passing through the swing doors a lobby is entered having on the left the patients' clothes store, the duty room or ward kitchen, with a larder, and a clinical room which communicates directly with the ward, and on the right a linen room and two single-bedded wards. All these adjuncts are well planned and equipped, the clinical room being especially good and having direct communication with the ward. Its equipment is remarkably complete, and here is placed the poisons cupboard, a perfect model, being an entirely new design, originating, we believe, with the matron, Miss Ray. A notable medicine cupboard, with the same origin, of special design and excellence, is placed in the ward.

### The Larger Wards Described.

Entering the ward, the special character of the windows at once strikes the eye, for they are entirely without boxing, sash lines, or weights, being arranged on the system of one sash balancing the other on a pivot, turning about a fixed point. A check is provided whereby the frames open to such an extent that ample central ventilation is provided with a minimum of draught. By moving, this check can be made to form hopper lights equal to opening the whole window area, and by releasing two catches each sash can be swung for cleaning. Above the windows are hopper ventilators with glazed cheeks reaching to the ceiling. The glass of the hoppers is obscured in order to diffuse direct sunlight, and enables the blind to be fixed at the transom level. We carefully tested these windows and studied the effect of the weather and the wear and tear upon them. We found that this test gave perfect results, and we commend this Austral pattern as a great improvement upon most other windows yet in use.

The Out-patients' Department, King's College Hospital.

The floors are of the Mouchel-Hennebique system of ferro-concrete, the space between the beams being occupied by hollow terra-cotta blocks; these fulfil the double purpose of preventing the conduction of sound—a most important point, as experience teaches—whilst they allow the ward ceilings to be plastered flush. Linoleum has been placed on the floors, which is laid on a Durato subfloor, with a Durato border and coved skirting. All internal angles are coved to a radius of 2½ inches. The effect and results of a year's trial justify confidence in their complete success.

The ward equipment and the heating and warming by radiant heat will be dealt with in separate articles on another occasion.

Two small bays, one on each side, are a novel feature. They contain basins for the use of the staff and shelves for sterilisers, lotion bowls, and so forth. The sanitary fittings to these basins should be studied and compared with those in the lavatories and elsewhere throughout the hospital, the first pattern being fixed and the others capable of removal for cleaning. The fixed ones have the disadvantage of being relatively difficult to clean, and lint and other undesirable things are apt to accumulate in the grating below the basin, which calls for additional watchfulness and attention by the nursing staff.

At the far end of the ward are large glass doors leading on to the sun balcony, and to the right and left are placed entrances to the sanitary towers through cut-off lobbies, the doors and partitions in which are fixed 9 inches clear of the terrazzo floor. A novel and excellent feature of the tower containing the bathroom is the introduction of a wash-up lavatory leading out of the passage through which the bathroom is reached. This excellent arrangement is followed throughout the hospital, the wash-up being placed in the case of the special ward block as part of the ward unit, at the extreme end of the lobby entrance to it, and has direct communication with the ward. These wash-up arrangements for the first time secure reasonable comfort and isolation for the nurses, and free them from surroundings and circumstances which in the old type of sanitary blocks were often as insanitary and unpleasant as they were condemnatory on moral and personal grounds. The terrazzo floor is made with channels into which the baths and basins discharge.

The tower, which is used wholly for sanitary purposes, contains an ample sink room admirably planned with excellent arrangements for heating bed-pans, for washing mackintoshes, for the preservation and examination of excreta and specimens, and for foul and soiled linen, with an admirable hermetically sealed trolley which enables these articles to be moved to the lift and sent to their proper place without the slightest inconvenience or other disadvantage or danger. The sink room is provided with close-fitting metal doors. Both these units deserve attention and study.

The wards are 108 feet 1½ inches long by 26 feet 3 inches wide and 13 feet high. They contain twenty-four beds, have a wall space of 8 feet 6 inches per bed, centre to centre, have a superficial area of 122 feet per bed, and a cubic capacity of 1,575 feet per bed. Each bed has a window on either side, the glass area being 1 foot super to 70 feet cube. The entrance doors to each ward are glazed with an ingenious combination of obscured and clear plate glass. The advantage claimed for this is that supervision of the ward from the corridor and *vice versa* is so made possible without opening these doors. Other doors are of flush hardwood, with double glazed flush bull's-eye lights where necessary.

It may here be noted that the main hospital corridor connects all the ward blocks on each floor, and that the principal staircase with the bed lift serves all these floors. There are subsidiary staircases and coal and diet lifts directly connected with the stores and kitchen. Another detail of importance is that a staircase with a bed lift connects each floor of the corridor on the north side and is used in conjunction with the operation theatres. Who shall estimate the amount of human suffering which may be avoided or the quality and extent of the risks which sometimes attend the removal of patients over long distances from an operation theatre to a ward, which this excellent plan should completely obviate and wholly prevent?

**The Entrance Hall, King's College Hospital.**

### Special Ward Pavilions.

No exact information is available as to all the special departments provided with wards in this block. The wards for ear and throat and skin patients, male and female, are, we know, provided on the first floor, the ophthalmic patients being, we believe, on the second floor, where the special operation theatre for these cases has a bed placed under the window so that the head of the patient can be brought immediately under the light. Noteworthy features of the special wards are that each contains fourteen beds, one ward being for men and the other for women. On each floor there is a special operation theatre with an examination and anæsthetising room. There is only one sanitary tower in connection with each ward, the bathrooms being incorporated with the ward unit proper. These bathrooms are situated in the centre of each pavilion, the two bathrooms, one for each ward, being entered from the ward. There is also for each division in the centre the usual ward unit adjuncts, including the duty room or ward kitchen, linen-room, larder, patients' clothes, and other accommodation.

### The Nursing Department and its Teaching School.

The nursing department throughout is one of the best planned and most complete productions of the kind we have seen. The administrative portion is on the first floor. The matron's office accommodation is excellent and includes a comfortable secretary's room and a waiting room, in addition to the office proper. Adjacent to it are a uniform-makers' room, three sisters' sitting rooms, and a sick nurses' dispensary and consulting room. The nurses have a fine recreation room, with a balcony over the entrance portico, from which there is an excellent view. There are also staff nurses' and probationers' sitting rooms and a writing room. At either end of the first floor are situated the sister-matron's and the assistant matron's apartments, really flats; the only difference in this excellent and attractive accommodation appears to be that the sister-matron has a dining room in addition to a sitting room, bedroom, bath, etc. Each has a small internal passage hall, and the flats present a restful, healthful atmosphere and freedom from official cares.

On the second floor are situated the nurses' bedrooms, the corridors leading to which are covered with linoleum. A bathroom is provided for every six bedrooms on an average, and there are disconnected sanitary towers on each floor. A basin is fitted in each bathroom, and the baths are so constructed as to permit of a limited but ample supply of hot water to be assured on each occasion of their use. Three hair-brushing rooms are provided, a feature of which is the effective protection against fire in connection with the drying apparatus. Each nurse's bedroom averages twelve feet by nine feet. It has a built-in wardrobe reaching to the ceiling, a marble angle slab for jug and basin, and a smaller one for other things. The corridors are warmed by radiators, and a fanlight with a check stay for each door regulates the entry of warm air, an arrangement which, in combination with an extract flue, secures the constant circulation of air.

Over the board room is placed the nurses' class-room, which is well lighted by a clerestory top light. It is provided with ample apparatus of all kinds, including a complete installation, with a range, of every requisite for cookery demonstrations and instruction. The assistant-matron in charge of the classroom, who is responsible for the nurses' tuition, has her office near at hand. All these arrangements are commendable and complete. They should make the new King's College Hospital nurse-training school one of the most effective and popular in the land.

### The Kitchen and Stores Department.

The kitchen is attractive and unusually large, measuring 70 feet by 35 feet, being situated in the courtyard between the east and centre blocks. A screened and ample service lobby separates it from the main hospital corridor (basement), whence access is obtained to the service lifts. The roof is worthy of examination, being constructed in ferro-concrete, and constitutes an interesting example of the use of this material.

Around the kitchen are placed the various stores and larders, sculleries, pastry rooms, and other accommodation, and the cook's office is planned to overlook the whole kitchen. Adjacent to the kitchen is the sister-housekeeper's office, and near at hand will be found the various grocery, hardware, and other stores, which have been fitted up upon a plan devised by Miss Ray, which presents some food for thought, for it has proved most suggestive and admirable in many ways. It is noteworthy that throughout this department there is an absence of all disagreeable smell, and that the free circulation of air is everywhere welcomely apparent. By a thoughtful arrangement of areas the kitchen is divided from the main blocks, and an ample number of flues successfully carry off all cooking smells. All the fittings in the main kitchen are Slaters' Ltd. They show much of this firm's best work, but leave something to desire in the type of boilers for steaming, which are fixed and by no means easy to clean, if it be even ever possible to make them present internally, when in use, the attractive features which every good cook sets her heart upon securing. Why should not Messrs. Slaters adopt the chef's experience and encourage a useful pride in smartness in connection with the boilers they supply by making them movable, so that they may afford every facility for thorough cleaning and the enforcement of complete efficiency and smartness?

### Kitchen Staff and Wardmaids' Accommodation.

The accommodation provided under this head at the new King's College Hospital presents some novel features, and is highly commendable both in regard to its quality and equipment. The servants are housed in the two upper floors of the central block; cubicle accommodation is provided for about fifty, with several larger bedrooms set apart for the upper servants. There is a reasonable supply of bathrooms and sanitary accommodation, but we have no support for large lavatories with a range of basins where several women may have to wash at the same time. Much thought and care have evidently been expended on this accommodation, and the servants no doubt feel themselves to be exceptionally well provided for. The system of checking in and checking out members of the staff is a feature of the management which should be noted by the visitor.

### Special Note.

We must once more insist that with 700 military and civil patients under treatment with a mixed system of administration in this new hospital it is not possible to do either the work or the administration full justice. When it becomes entirely a civil hospital it will be practicable to deal with the technical administrative and other departments in their working dress under normal conditions. We confess our disappointment that the great war has deprived London of the immediate and continuous use by the civil population of this most interesting group of modern hospital buildings.

## Figures and Tables

**Figure f1:**
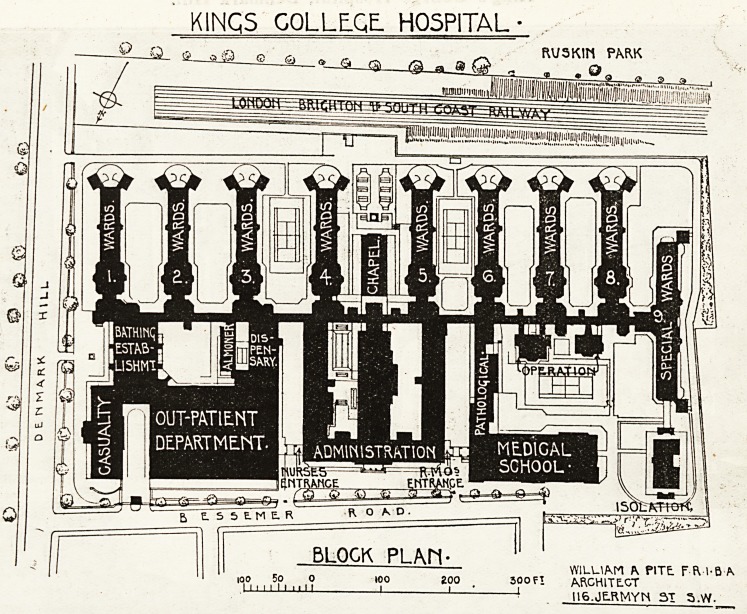


**Figure f2:**
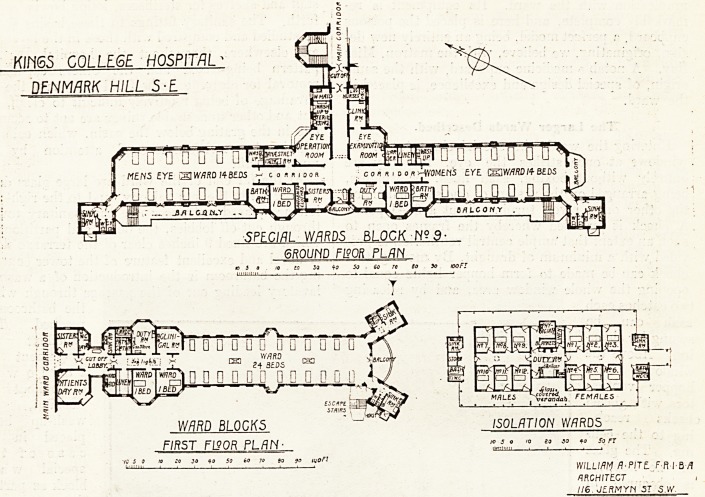


**Figure f3:**
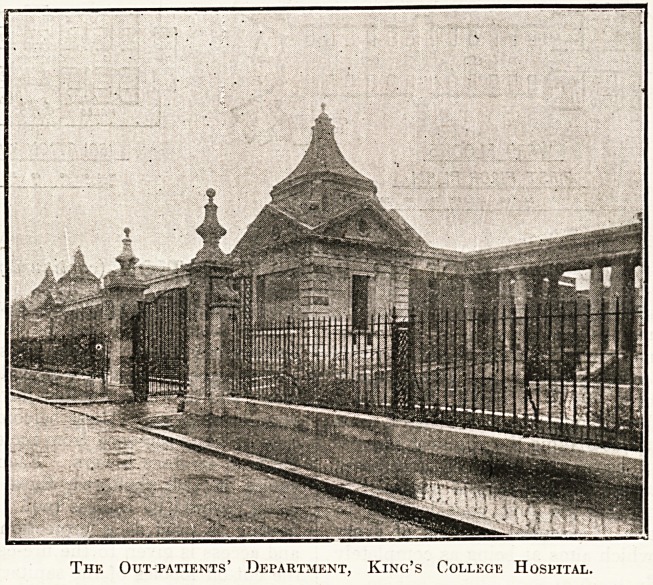


**Figure f4:**